# Supplementary material for: The absence of orthostatic heart rate increase is associated with cognitive impairment in Parkinson’s disease
Source: PLoS One. 2020 Oct 15;15(10):e0240491. doi: 10.1371/journal.pone.0240491 (PMC7561175; doi:10.1371/journal.pone.0240491)
Supplement: Attachment — Submitted filename: Response to Reviewers PLOS ONE [PONE-D-20-22293].docx [file pone.0240491.s001.docx]

**Response to reviewers,**

*Additional Editor Comments (if provided):*

*The authors retrospectively analyzed 147 cases of clinically diagnosed PD to determine the association between the absence of a heart rate response and cognitive impairment in PD with OH. Among the patients with OH, neurogenic OH was diagnosed in cases without a heart rate increase, while all other patients were diagnosed with nonneurogenicOH. Dementia was found in 25 of 147 PD cases (17%) in this cohort. The presence of OH was an independent risk factor for dementia in PD in addition to the disease severity and years of education. Neurogenic OH was significantly associated with dementia compared to the no OH group (harzard ratio [HR] 8.2, 95% confidence interval [CI] 2.5-26.9, P<0.001), an association that was preserved after adjusting for age, gender and other covariant factors. However, no such association was observed for non-neurogenic OH (HR 2.7, 95%CI 0.7-10.5, P=0.13). While the cognitive impairment was significantly worse in the neurogenic OH group than the no-OH group, the groups were otherwise similar. The blood pressure decrease was significantly lower in both OH groups than in the no-OH group, despite no significant differences between the OH groups.
The manuscript is interesting. However, I have a concern regarding the presence of syncope and/or unexplained falls in this sample. Please see and discuss Ungar A: Etiology of Syncope and Unexplained Falls in Elderly Adults with Dementia: Syncope and Dementia (SYD) Study. J Am Geriatr Soc. 2016 Aug;64(8):1567-73.*

**Response:**

We thank the editor for the important suggestion concerning the presence of syncope and unexplained fall in this study. Unfortunately, we did not collect information on syncope or unexplained fall in this study, so we could not analyze these issues. However, as the editor indicated, whether or not syncope and unexplained fall are risks for dementia and the relationship between orthostatic hypotension and syncope or unexplained fall in PD are clinically important issues. We have now mentioned this in the Discussion of the revised manuscript (page 18, line 267-271, page 198, line 272).

*Reviewer #1: The paper is of interest and the association between orthostatic hypotension (OH) , cognitive impairment and Parkinson’s disease (PD) is clinically relevant. The association between the heart rate response and cognitive impairment was retrospectively analyzed in 147 cases of clinically diagnosed PD to determine the association between the absence of a heart rate response and cognitive impairment in PD with OH. Among the patients with OH, neurogenic OH was diagnosed in caseswithout a heart rate increase, while all other patients were diagnosed with non-neurogenic OH. Dementia was found in 25 of 147 PD cases (17%). Neurogenic OH was significantly associatedwith dementia compared to the no OH group (harzard ratio [HR] 8.2, 95% confidenceinterval [CI] 2.5-26.9, P<0.001), an association that was preserved after adjusting forage, gender and other covariate. No association was observedfor non-neurogenic OH (HR 2.7, 95%CI 0.7-10.5, P=0.13). The manuscript is well written, data support conclusions.*

**Rensponse:**

We thank the reviewer for this positive evaluation. In accordance with the comment from the academic editor and Reviewer 2, we modified the manuscript accordingly.

*Reviewer #2: The paper is interesting, resulting in a great impact on management of PD patients. The correlation of dementia in PD patients with neurogenic OH is a relevant finding to better understand evolution and prognosis of PD. However, some aspects should be more extensively argued to avoid confounding data.

Major revision
Materials and methods: In this study, drugs were not considered with exception of anti-hypotensive medications and levodopa. The influence of some classes of medications on postural response of arterial pressure is well known. In particular, there is no mention about beta-blockers. Patients with congestive heart failure were excluded from the study, but use of beta-blockers is also and often requested in hypertensive patients or in coronary artery disease (enrolled in this study). This class of medications could fade postural heart rate response leading to a wrong diagnosis of neurogenic OH.
The study should be improved by adding data on medications to avoid that the association of neurogenic OH with dementia in PD patients is considered only a winsome suggestion.*

**Response:**

First, we reviewed the data and found four patients had been treated with midodrine during the investigation. We apologize for our mistake, and those four patients have been excluded from this study. As the reviewer suggested, we reviewed the data again and added the information on anti-hypertensive medications such as angiotensin-converting-enzyme inhibitors (ACE-Is), angiotensin II receptor blockers (ARBs), calcium channel blockers (CCBs), beta blockers, and diuretics. There were no patients who were treated with ACE-Is. There were no significant differences in the prevalence of the use of any anti-hypertensive medications, except for beta-blockers, between PD and PDD (Table 1). Beta-blocker use was found in only four patients, including two patients who used these agents for hypertension and another two patients who used them for tremor. The basal results showed a similar trend to the original data; however, beta-blocker use was significantly associated with PDD (Table 2), and the ORs for dementia of non-neurogenic OH were significant after adjusting for beta-blocker use (Table 3). Because the number of patients prescribed beta-blockers in this study was very small, we were unable to draw any conclusion regarding whether or not beta-blocker use is a risk factors significantly associated with dementia in PD. Further, large-scale clinical studies will be needed to clarify this issue.

We have now revised these results in the Abstract (page 3), Methods (page 7, line 87, line 90-93), and Results (page 10, line 140, line 146-147, page 11, line 151, line 154-157, page 12, line 167-174, page 13, line 185-187) sections and all tables. We also discussed this in the Discussion section (page 14, line 198, page 15, line 201, page 18, line 257-263, line 267-271, page 19, line 272).
